# Hazardous Effects of Curcumin on Mouse Embryonic Development through a Mitochondria-Dependent Apoptotic Signaling Pathway

**DOI:** 10.3390/ijms11082839

**Published:** 2010-08-02

**Authors:** Chia-Chi Chen, Ming-Shu Hsieh, Yan-Der Hsuuw, Fu-Jen Huang, Wen-Hsiung Chan

**Affiliations:** 1 Department of Bioscience Technology and Center for Nanotechnology, Chung Yuan Christian University, Chung Li, Taiwan; E-Mails: cha_chi_chen@yahoo.com.tw (C.-C.C.); virgilhsieh@yahoo.com.tw (M.-S.H.); 2 Department of Life Science, National Pingtung University of Science and Technology, Pingtung, Taiwan; E-Mail: hsuuw@yahoo.com.tw; 3 Department of Obstetrics and Gynecology, Chang Gung Memorial Hospital-Kaohsiung Medical Center and Chang Gung University College of Medicine, Taiwan

**Keywords:** curcumin, blastocyst, apoptosis, development, ROS

## Abstract

In this study, we examined the cytotoxic effects of curcumin, the yellow pigment of *Curcuma longa*, on the blastocyst stage of mouse embryos, subsequent embryonic attachment, and outgrowth *in vitro* and *in vivo* implantation by embryo transfer. Mouse blastocysts were incubated in medium with or without curcumin (6, 12 or 24 μM) for 24 h. Cell proliferation and growth were investigated using dual differential staining, apoptosis was analyzed with terminal deoxynucleotidyl transferase-mediated dUTP nick-end labeling (TUNEL), and implantation and post-implantation development of embryos were measured by *in vitro* development analysis and *in vivo* embryo transfer, respectively. Blastocysts treated with 24 μM curcumin displayed significantly increased apoptosis and decreased total cell number. Interestingly, we observed no marked differences in the implantation success rates between curcumin-pretreated and control blastocysts during *in vitro* embryonic development through implantation with a fibronectin-coated culture dish. However, *in vitro* treatment with 24 μM curcumin was associated with decreased implantation rate and increased resorption of postimplantation embryos in mouse uterus, as well as decreased fetal weight in the embryo transfer assay. Our results collectively indicate that *in vitro* exposure to curcumin triggers apoptosis and retards early postimplantation development after transfer to host mice. In addition, curcumin induces apoptotic injury effects on mouse blastocysts through ROS generation, and further promotes mitochondria-dependent apoptotic signaling processes to impair sequent embryonic development.

## 1. Introduction

Curcumin, a common dietary pigment and spice, is a hydrophobic polyphenol derived from the rhizome of the herb *Curcuma longa* that is used as a traditional Indian medicine [1] for the treatment of wounds, liver ailments, hepatitis and urinary tract diseases, as well as a cosmetic compound [2]. Curcumin exerts a wide range of pharmacological effects, including anti-inflammatory, anti-carcinogenic, hypocholesterolemic and anti-infection activities [3–8]. As a potential antioxidant, curcumin displays anti-proliferative and anti-carcinogenic properties in a variety of cell lines and animals [8–12]. Moreover, the efficacy of curcumin in various diseases, including cancer, is well established [13]. Recent studies have shown that the anti-tumor activity of curcumin is attributed to its ability to induce apoptosis via caspase-3 activation [14,15]. Moreover, various *in vivo* animal assay models and human studies confirm that dietary curcumin is extremely safe and does not exert hazardous effects, even at high doses [16–19]. For example, three separate phase I clinical trials demonstrate that dietary curcumin administered at doses as high as 12 g per day is well tolerated [18–20]. Curcumin displays high pharmacological safety and efficacy, and is thus a potential candidate agent for the treatment and prevention of a wide range of human diseases. Importantly, a recent study by our group shows that curcumin inhibits methylglyoxal-induced reactive oxygen species (ROS) generation and various apoptotic biochemical events in embryonic stem cells and blastocysts isolated from pregnant mice [21]. Moreover, another study by our group focusing on the possible effects of curcumin on ROS generation, intracellular adenosine triphosphate (ATP) levels and cell death mode in osteoblast cells revealed that curcumin induces apoptosis or necrosis in a dose-dependent manner [15]. However, while multiple biological functions have been identified for curcumin, the ambiguous issue of its activity as an apoptotic inducer or inhibitor and the precise molecular mechanisms underlying these actions are yet to be fully determined. To date, virtually no studies have investigated the potential of curcumin as a cytotoxic agent against embryo development.

Apoptosis plays important roles in development and disease [22]. While apoptosis is an established contributor to normal embryonic development [23–25], several other studies have shown that mechanistically diverse teratogens induce excessive apoptosis in early embryos, leading to developmental impairment [21,26–30]. Importantly, a recent investigation by our group revealed that curcumin induces apoptotic changes, including c-Jun N-terminal kinase (JNK) activation, caspase-3 activation, and cleavage of poly-(ADP-ribose) polymerase (PARP) and p21-activated kinase 2 (PAK2) at treatment concentrations less than 25 μM in human osteoblast cells. In contrast, 50–200 μM curcumin did not induce apoptosis but triggered necrotic cell death in human osteoblasts [15]. In a further study, the curcumin dosage was show to determine its possible effects on ROS generation, intracellular ATP levels, and apoptosis or necrosis in osteoblast cells [15]. These findings collectively indicate that curcumin promotes apoptosis or necrosis in a dose-dependent manner in human osteoblast cells. To our knowledge, the present report is the first to show that the curcumin dosage significantly influences the cell death mode of osteoblasts. These novel findings provide important insights into the impact of curcumin on other mammalian cell lines, particularly in terms of embryonic stem cells or embryonic development.

Here, we examined whether curcumin has cytotoxic effects and determined the related regulatory mechanisms in mouse embryonic development. Our results show that curcumin suppresses embryonic cell proliferation during the blastocyst stage predominantly by inducing apoptosis in the inner cell mass (ICM). We also monitored subsequent impairment of blastocyst development *in vitro* and following embryo transfer *in vivo*. However, the mechanisms underlying curcumin-induced apoptosis of mouse blastocysts remain to be determined.

## 2. Results and Discussion

**Figure 1:**
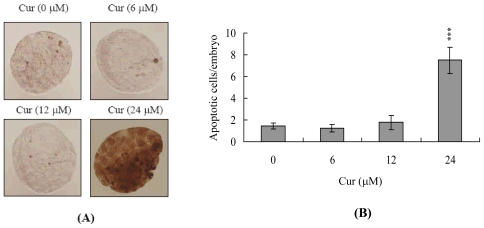
Curcumin induces apoptosis in mouse blastocysts. (**A**) Mouse blastocysts were treated with curcumin (Cur; 6, 12 or 24 μM) for 24 h or left untreated, and apoptosis examined using TUNEL staining. The results were visualized using light microscopy. TUNEL-positive cells are depicted in black. (**B**) The mean number of apoptotic (TUNEL-positive) cells per blastocyst was calculated as five to eight determinations. Values are presented as means ± SEM. *** P < 0.001 *versus* the control group.

To initially examine the possibility of curcumin-induced cytotoxicity, we treated mouse blastocysts with 6, 12 or 24 μM curcumin at 37 °C for 24 h, and monitored apoptosis using the TUNEL method. Curcumin clearly induced apoptosis in mouse blastocysts at a concentration of 24 μM (Figure 1A). Quantitative analysis revealed 7.5-fold higher apoptosis in curcumin-treated blastocysts *versus* untreated controls (Figure 1B), confirming curcumin-induced apoptosis in mouse blastocysts.

**Figure 2:**
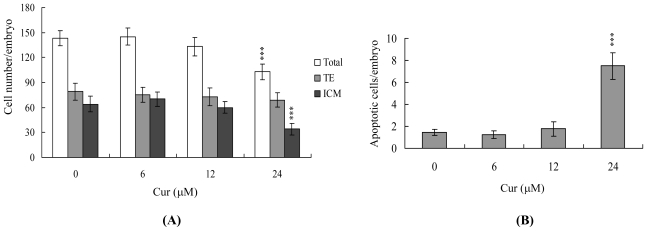
Effects of curcumin on blastocyst viability. Mouse blastocysts were treated with curcumin (Cur; 6, 12 or 24 μM) for 24 h or left untreated. **(A)** The total number of cells per blastocyst and cell numbers in the inner cell mass (ICM) and trophectoderm (TE) were counted. **(B)** The percentages of Annexin V-positive/PI-negative cells in the blastocysts of each group were examined. Data are based on at least 250 blastocyst samples from each group. Values are presented as means ± SEM of six determinations. *** P < 0.001 *versus* the control group.

We further used differential staining for cell counting to examine proliferation in blastocysts treated with 6, 12 or 24 μM curcumin or left untreated for 24 h. The results revealed significantly lower total and ICM cell numbers in curcumin-treated blastocysts (24 μM) *versus* controls (Figure 2A). Annexin V and PI staining were further used to detect the cell death modes in ICM or TE or both. We observed significantly higher numbers of Annexin V-positive/PI-negative (apoptotic) cells in the ICM of treated blastocysts *versus* controls, but no differences in the TE (Figure 2B). Thus, it seems that curcumin induces significant apoptosis in the ICM but not TE of mouse blastocysts. These results further indicate that curcumin impairs the developmental potential of blastocysts by inducing ICM apoptosis.

**Figure 3:**
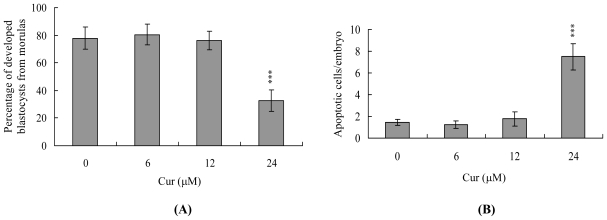
*In vitro* development of mouse embryos exposed to curcumin at the blastocyst stage. **(A)** Mouse morulas were treated with curcumin (Cur; 6, 12 or 24 μM) for 24 h or left untreated, and cultured for an additional 24 h at 37 °C. Blastocysts were counted and percentages calculated. **(B)** Mouse blastocysts were treated with curcumin (Cur, 6, 12 or 24 μM) for 24 h or left untreated, and observed in culture for 7 days post-treatment. Blastocysts were identified as attachment only, ICM (+), ICM (++), and ICM (+++) via morphological assessment, as described in Materials and Methods. Values are presented as means ± SEM of eight determinations. *** *P* < 0.001 *versus* the control group.

We further analyzed the effects of curcumin on embryonic preimplantation, implantation and postimplantation development *in vitro*. Untreated control morulas displayed ~78% development into blastocysts, whereas only 32.5% of the 24 μM curcumin-treated morulas developed into blastocysts under our *in vitro* experimental conditions (Figure 3A). To analyze the effects of curcumin on implantation and postimplantation events *in vitro*, blastocysts were treated with 6, 12 or 24 μM curcumin (250–300 blastocysts for each group) or left untreated, and the implantation rate and subsequent development for 8 days in culture were analyzed. The implantation rate of and attachment to the fibronectin-coated dish were similar in the curcumin-treated group and the untreated control group (Figure 3B). Importantly, curcumin-pretreated blastocysts displayed a lower incidence of postimplantation developmental milestones compared with control blastocysts (Figure 3B). The results indicate that curcumin affects the *in vitro* potential of blastocysts to develop features of postimplantation embryos.

**Figure 4:**
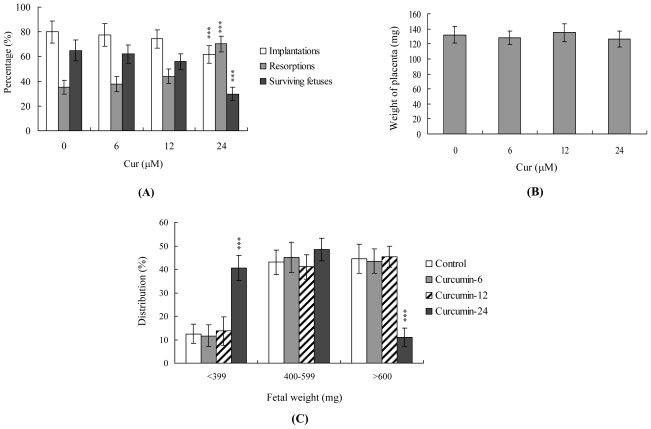
Effects of curcumin on mouse blastocysts on *in vivo* implantation, resorption, fetal survival and fetal weight. **(A)** Mouse blastocysts were treated with curcumin (Cur, 6, 12 or 24 μM) for 24 h or left untreated. Implantations, resorptions and surviving fetuses were analyzed, as described in Materials and Methods. The percentage of implantations represents the number of implantations per number of transferred embryos × 100. The percentage of resorptions or surviving fetuses denotes the number of resorptions or surviving fetuses per number of implantations × 100. **(B)** Placental weights of 40 recipient mice were measured. **(C)** Weight distribution of surviving fetuses on day 18 post-coitus. Surviving fetuses were obtained by embryo transfer of control and curcumin-pretreated blastocysts, as described in Materials and Methods (320 total blastocysts across 40 recipients). *** P < 0.001 *versus* the curcumin-free group.

To further determine the effects of curcumin on blastocyst development *in vivo*, we transferred mouse blastocysts subjected to curcumin pretreatment or left untreated, and examined the uterine content at 13 days post-transfer (day 18 post-coitus). The implantation ratio in the curcumin-pretreated group (24 μM) was significantly lower than that of the untreated control group (Figure 4A). In addition, embryos that implanted but failed to develop were subsequently resorbed. Early resorption was visualized as small dark moles with no distinct structures, whereas late resorptions resembled placentas with no fetal structure. The proportion of implanted embryos that failed to develop normally (early and late resorption groups) was significantly higher in the 24 μM curcumin-pretreated group *versus* the control group (Figure 4A). Interestingly, no differences in placental weight were evident between the curcumin-treated and untreated groups (Figure 4B), but fetal weight was lower in the curcumin-treated group. Moreover, previous and recent experiments by our group reveal that 35–40% of fetuses weigh more than 600 mg, with the average weight of total surviving fetuses being ~600 ± 12 mg in the untreated control group at day 18 of pregnancy in a mouse embryo transfer assay [27,31–34]. Fetal weight is an important indicator of developmental status, and the average fetal weight of the untreated control group was thus used as a key indicator for measuring the development status of curcumin-treated blastocysts. The results show that only about 11% of the fetuses in the 24 μM curcumin-pretreated group weighed more than 600 mg (an indicator of successful embryonic and fetal development), in contrast to 44% of control fetuses (Figure 4C). Thus, curcumin exposure at the blastocyst stage poses a potential risk to postimplantation development.

**Figure 5:**
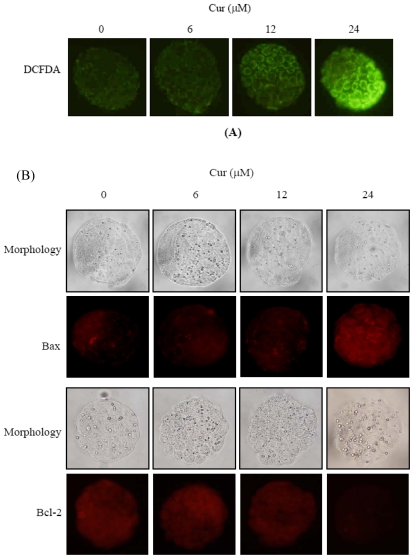

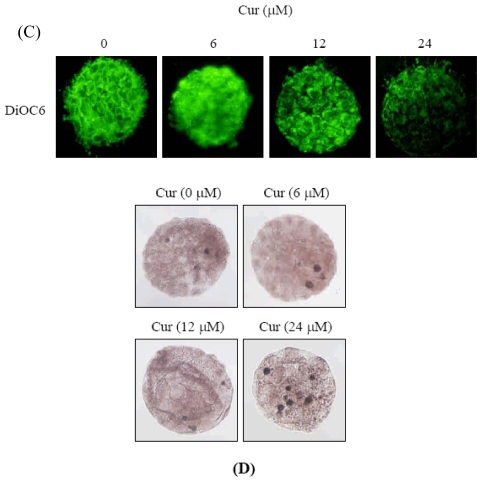
Effects of curcumin on ROS generation and mitochondria-dependent apoptotic processes in mouse blastocysts. Mouse blastocysts were treated with curcumin (Cur, 6, 12 or 24 μM) or left untreated for 24 h. **(A)** ROS generation was detected by staining with 20 μM DCF-DA fluorescence dye. **(B)** Bax and Bcl-2 expression levels were determined by immunostaining with anti-Bax and anti-Bcl-2 antibodies, respectively. The protocol is described in “Materials and Methods”. **(C)** To examine mitochondrial membrane potential changes, embryos were incubated with 40 nM DiOC6(3) at 37 °C for 1 h and analyzed under a fluorescence microscope. **(D)** Activation of caspase-3 was analyzed by immunostaining with anti-activated caspase-3 antibody for 3 h, followed by a secondary antibody conjugated with peroxidase (1:100) for 1 h. Finally, 20 μL of DAB-substrate solution was added to embryos, and incubated for 2 min at room temperature. Cells with activated caspase-3 are presented in black.

In light of previous reports and our recent finding that ROS effectively induces apoptosis [27,35,36], we further used the fluorescent dye DCF-DA to measure the ROS content in curcumin-treated mouse blastocyst cells. As shown in Figure 5A, 24 μM curcumin directly induced an increase in fluorescence intensity in mouse blastocysts, compared with untreated control cells. Expression changes in Bax and Bcl-2 are additionally relevant to the mitochondria-dependent apoptotic pathway [37,38], with high and low Bax/Bcl-2 ratios associated with lower and higher apoptotic thresholds, respectively. Accordingly, we determined whether curcumin induces apoptosis via modulation of Bax and Bcl-2 expression. Immunostaining experiments revealed increases and decreases in the Bax and Bcl-2 levels, respectively, upon curcumin treatment of mouse blastocysts (Figure 5B). Further investigation of the effects of curcumin on mitochondrial membrane potential (MMP) changes in mouse blastocyst cells revealed that treatment with 24 μM curcumin suppressed DiOC_6_(3) uptake into the mitochondria of mouse blastocyst cells, indicative of significant MMP loss (Figure 5C). In addition, 24 μM curcumin significantly stimulated caspase-3 activation, an important indicator of apoptosis (Figure 5D).

**Figure 6:**
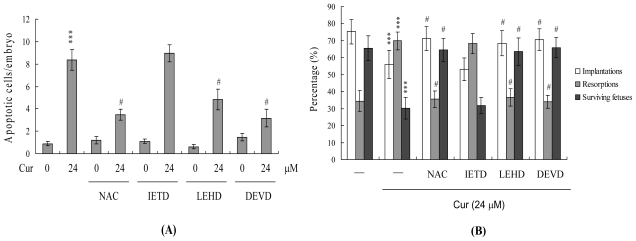

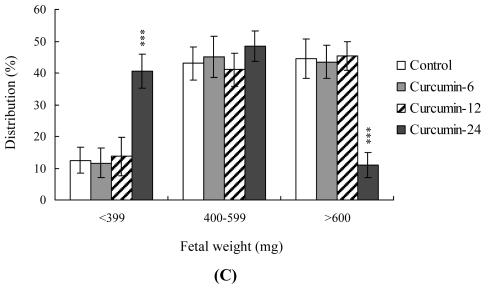
Effects of ROS scavengers and caspase inhibitors on *in vivo* implantation, resorption, fetal survival and fetal weight in curcumin-treated embryos. Mouse blastocysts were pretreated with 400 μM N-acetyl cysteine (NAC), 300 μM Z-IETD-FMK (IETD), 300 μM Z-LEHD-FMK (LEHD) or 300 μM Z-DEVD-FMK (DEVD) for 1 h or left untreated. Blastocysts were further incubated with curcumin (Cur, 24 μM) for another 24 h. (**A**) Apoptosis was examined using TUNEL staining, as described in Figure 1. (**B**) Implantations, resorptions and surviving fetuses were analyzed by embryo transfer, as described in Materials and Methods and Figure 4. (**C**) The weight distribution of surviving fetuses on day 18 post-coitus. Surviving fetuses were obtained by embryo transfer of control and curcumin-pretreated blastocysts (320 total blastocysts across 40 recipients). *** P < 0.001 *versus* the curcumin-free group and #P < 0.001 *versus* 24 μM

To further determine the role of ROS and apoptotic associated events in curcumin-induced apoptosis, we assessed the effects of a commonly used ROS scavenger, N-acetyl cysteine (NAC), as well as various caspase-specific inhibitors on curcumin-treated mouse blastocysts. Pretreatment of cells with NAC (500 μM) attenuated curcumin-induced apoptosis (Figure 6A). Moreover, pretreatment with caspase-9 (Z-LEHD-FMK) and caspase-3 (Z-DEVD-FMK) specific inhibitors effectively blocked apoptosis, while the caspase-8 inhibitor Z-IETD-FMK had no effect (Figure 6A). Importantly, treatment with 24 μM curcumin was associated with a lower implantation ratio, and failure of further development was effectively blocked by NAC and inhibitors of caspase-9 and caspase-3 by embryo transfer. The group pretreated with the caspase-8 inhibitor displayed no significant differences compared with the untreated control (Figure 6B). In addition, fetal weight was lower in the 24 μM curcumin-treated group, which was effectively rescued upon pretreatment with NAC and specific caspase-9 and -3 inhibitors (Figure 6C). Based on these data, we suggest that curcumin triggers ROS generation, in turn, activating mitochondria-dependent apoptotic processes in mouse blastocyst cells.

We have previously shown that curcumin induces osteoblast apoptosis at doses of 12.5–25 μM and necrosis at doses greater than 50 μM [15]. Interestingly, curcumin is able to both stimulate and inhibit apoptotic signaling. For instance, curcumin induces apoptosis in human melanoma (30–60 μM for 24 h) [39], human leukemia HL 60 (10–40 μM for 16–24 h) [40,41], AK-5 tumor (10 μM for 18 h) [14,42] and MCF-7 breast cancer cells (25_ μM for 24 h) [43]. Conversely, curcumin (10 μM for 12 h) inhibits dexamethane-induced apoptosis in rat thymocytes, chemotherapy-induced apoptosis in breast cancer cells [44,45], and methylglyoxal-triggered apoptosis in mouse embryonic stem cells [21]. Our novel results, along with previously published findings, indicate that the treatment protocol (*i.e.*, treatment period and dosage) determines the effects of curcumin on various cell types. Moreover, during the complex and precisely orchestrated process of embryonic development, chemical or physical injury can affect normal development and lead to malformation or miscarriage of the embryo. Thus, it is important to examine the possible teratogenic effects of various agents, including natural chemical compounds contained in food that are significant potential apoptotic inducers, such as curcumin. In the present study, we investigated whether curcumin adversely affects the blastocyst stage of mouse embryos and subsequent early postimplantation embryonic development. We report for the first time that treatment with 24 μM curcumin for 24 h induces apoptosis in mouse blastocysts (Figure 1). Based on this finding, we further analyzed the effects of curcumin on embryonic development by incubating blastocysts in medium containing 6, 12 or 24 μM curcumin for 24 h. Our results show that curcumin suppresses cell viability in mouse blastocysts via apoptosis (Figures 1 and 2). Dual differential staining further showed that this curcumin-induced cell loss and apoptosis occurs primarily in the ICM (Figure 2).

The TE arises from the trophoblast at the blastocyst stage and develops into a sphere of epithelial cells surrounding the ICM and blastocoel. These cells contribute to the placenta and are required for development of the mammalian conceptus [46], indicating that reduction in the TE cell lineage may reduce implantation and embryonic viability [47,48]. However, in our experiments, curcumin induced cell apoptosis only in the ICM and not TE, and did not have deleterious effects on embryonic attachment and outgrowth *in vitro*. Importantly, we observed a lower implantation rate of curcumin-pretreated embryos in the mouse uterus compared with the untreated control group *in vivo* using the embryo transfer assay (Figures 2–4). Previous studies have shown that a ~30% or greater reduction in the number of cells in the ICM is associated with high risk of fetal loss or developmental injury, even in cases where the implantation rate and TE cell numbers are normal [49]. In addition, the ICM cell number is essential for proper implantation, and reduction in this cell lineage may decrease embryonic viability [47,48,50]. While apoptosis is responsible for eliminating unwanted cells during normal embryonic development, this process does not normally occur at the blastocyst stage [51,52]. Excessive apoptosis before or during the blastocyst stage is likely to delete important cell lineages, affecting embryonic development and potentially leading to miscarriage or embryonic malformation [53]. Thus, in view of the observation that curcumin reduces the cell number and increases apoptosis specifically in the ICM of mouse blastocysts, we investigated the possibility that curcumin causes embryonic implantation and mortality and/or developmental delay in postimplantation mouse embryos *in vitro* and *in vivo* (Figure 2). Our results show that curcumin-treated blastocysts suffer from decreased embryonic development and increased embryonic death *in vitro* and implantation *in vivo* (Figures 3 and 4).

Mechanistically, our data emphasize that curcumin directly evokes intracellular oxidative stress (Figure 5A), leading to ROS-mediated apoptosis in mouse blastocyst cells (Figure 6A). In addition, these effects seem to involve the mitochondria-dependent apoptotic pathway, as evident from curcumin-induced changes in the intracellular levels of Bcl family members (Bax and Bcl-2) and loss of mitochondrial membrane potential (Figure 5B). Our findings are consistent with previous data showing that curcumin induces apoptosis via activation of the mitochondrial pathway in human osteoblasts [15]. Given that recent studies have shown that the addition of specific compounds to commonly used cell culture media triggers generation of ROS, such as hydrogen peroxide [54,55], we co-incubated curcumin and culture medium, and measured ROS using the ferrous iron oxidation-xylenol orange method [54]. No artifactual ROS generation was detected under these conditions (data not shown). Importantly, a well-known ROS scavenger, NAC, effectively prevented curcumin-induced apoptosis in mouse blastocysts (Figure 6A). We further determined the precise mechanisms of curcumin-triggered apoptosis in mouse blastocysts (Figures 5 and 6). Our results collectively demonstrate that curcumin triggers apoptosis in ICM cells of mouse blastocysts, leading to impairment of embryo development via ROS generation, which stimulates downstream processes through the mitochondria-dependent pathway.

## 3. Experimental Section

### 3.1. Chemicals

Curcumin, Pregnant mare’s serum gonadotropin (PMSG), Bovine serum albumin (BSA), sodium pyruvate and puerarin were purchased from Sigma (St. Louis, MO, USA). Human chorionic gonadotropin (hCG) was obtained from Serono (NV Organon Oss, the Netherlands). The TUNEL *in situ* cell death detection kit was obtained from Roche (Mannheim, Germany) and CMRL-1066 medium was from Gibco Life Technologies (Grand Island, NY, USA). Z-DEVD-FMK, Z-LEHD-FMK and Z-IETD-FMK were from Calbiochem (La Jolla, CA, USA).

### 3.2. Collection of Mouse Morulas and Blastocysts

ICR mice were from National Laboratory Animal Center (Taiwan, ROC). This research was also approved by the Animal Research Ethics Board of Chung Yuan Christian University (Taiwan, ROC). All animals received humane care, as outlined in the Guidelines for Care and Use of Experimental Animals (Canadian Council on Animal Care, Ottawa, 1984). All mice were maintained on breeder chow (Harlan Teklad chow) with food and water available *ad libitum*. Housing was in standard 28 cm × 16 cm × 11 cm (height) polypropylene cages with wire-grid tops and kept under a 12 h day/12 h night regimen. Nulliparous females (6–8 weeks old) were superovulated by injection of 5 IU PMSG followed 48 hours later by injection of 5 IU hCG, and then mated overnight with a single fertile male of the same strain. The day a vaginal plug was found was defined as day 0 of gestation. Plug-positive females were separated for experimentation. Morulas were obtained by flushing the uterine tubes on the afternoon of gestation day 3, and blastocysts were obtained by flushing the uterine horn on day 4; in both cases the flushing solution consisted of CMRL-1066 culture medium containing 1 mM glutamine and 1 mM sodium pyruvate. The concentration of glucose in this medium was 5 mM. Expanded blastocysts from different females were pooled and randomly selected for experiments.

### 3.3. Curcumin Treatment and TUNEL Assay

Blastocysts were incubated in medium containing with or without 6, 12, or 24 μM curcumin for 24 h. For apoptosis detection, embryos were washed in curcumin-free medium, fixed, permeabilized and subjected to TUNEL labeling using an *in situ* cell death detection kit (Roche Molecular Biochemicals, Mannheim, Germany) according to the manufacturer’s protocol. Photographic images were taken under brightfield illumination using a fluorescence microscope.

### 3.4. Curcumin Treatment and Cell Proliferation

Blastocysts were incubated with or without culture medium containing indicated concentrations of curcumin (6–4 μM) for 24 h. And then, blastocysts were washed with curcumin-free medium and dual differential staining was used to facilitate counting of cell numbers in the inner cell mass (ICM) and trophectoderm (TE) [47]. Blastocysts were incubated in 0.4% pronase in M_2_–BSA medium (M_2_ medium containing 0.1% bovine serum albumin) for removal of the zona pellucida. The denuded blastocysts were exposed to 1 mM trinitrobenzenesulphonic acid (TNBS) in BSA-free M_2_ medium containing 0.1% polyvinylpyrrolidone (PVP) at 4 °C for 30 min, and then washed with M_2_ medium (Sigma) [56]. The blastocysts were further treated with 30 μg/mL anti-dinitrophenol-BSA complex antibody in M_2_-BSA at 37 °C for 30 min, and then with M_2_ medium supplemented with 10% whole guinea-pig serum as a source of complement, along with 20 μg/mL bisbenzimide and 10 μg/mL propidium iodide (PI), at 37 °C for 30 min. The immunolysed blastocysts were gently transferred to slides and protected from light before observation. Under UV light excitation, the ICM cells (which take up bisbenzimidine but exclude PI) appeared blue, whereas the TE cells (which take up both fluorochromes) appeared orange-red. Since multinucleated cells are not common in preimplantation embryos [57], the number of nuclei was considered to represent an accurate measure of the cell number.

### 3.5. Annexin V Staining

Blastocysts were incubated in 0, 6, 12 or 24 μM curcumin for 24 h, washed with curcumin-free culture medium, and then stained using an Annexin V-FLUOS staining kit (Roche), according to the manufacturer’s instructions. Briefly, the blastocysts were incubated in M_2_-BSA for removal of the zona pellucida, washed with PBS plus 0.3% BSA, and then incubated for 60 min with a mixture of 100 μL binding buffer, 1 μL fluorescein isothiocyanate (FITC)-conjugated Annexin V and 1 μL PI. After incubation, the embryos were washed and photographed using a fluorescence microscope under fluorescent illumination. Cells staining Annexin V+/PI- were considered apoptotic, while those staining Annexin V+/PI+ were considered necrotic.

### 3.6. Morphological Analysis of Embryonic Development

Blastocysts were cultured according to a modification of the previously reported method [58]. Briefly, embryos were cultured in 4-well multidishes at 37 °C. For group culture, four embryos were cultured per well. The basic medium consisted of CMRL-1066 supplemented with 1 mM glutamine and 1 mM sodium pyruvate plus 50 IU/mL penicillin and 50 mg/mL streptomycin (hereafter called culture medium). For treatments, the embryos were incubated with 0, 6, 12 or 24 μM curcumin for 24 h. Thereafter, the embryos were cultured for 3 days in culture medium supplemented with 20% fetal calf serum, and for 4 days in culture medium supplemented with 20% heated-inactivated human placental cord serum, for a total culture time of 8 days from the onset of treatment. Embryos were inspected daily under a phase-contrast dissecting microscope, and developmental stages were classified according to established methods [59,60]. Under these culture conditions, each hatched blastocyst attached to the fibronectin and grew to form a cluster of ICM cells over the trophoblastic layer via in a process called TE outgrowth. After a total incubation period of 96 h, morphological scores for outgrowth were estimated. Growing embryos were classified as either ‘attached’ or ‘outgrowth’, with the latter defined by the presence of a cluster of ICM cells over the trophoblastic layer. As described previously [61], ICM clusters were scored according to shape, ranging from compact and rounded ICM (+++) to a few scattered cells (+) over the trophoblastic layer.

### 3.7. Blastocyst Development Following Embryo Transfer

To examine the ability of expanded blastocysts to implant and develop *in vivo*, the generated embryos were transferred to recipient mice. ICR females (white skin color) were mated with vasectomized males (C57BL/6J; black skin color; from National Laboratory Animal Center, Taiwan, ROC) to produce pseudopregnant dams as recipients for embryo transfer. To ensure that all fetuses in the pseudopregnant mice came from embryo transfer (white color) and not from fertilization by C57BL/6J (black color), we examined the skin color of the fetuses at day 18 post-coitus. To assess the impact of curcumin on postimplantation growth *in vivo*, blastocysts were exposed to 0, 6, 12 and 24 μM curcumin for 24 h, and then 8 embryos were transferred in parallel to the paired uterine horns of day 4 pseudopregnant mice. The surrogate mice were killed on day 18 post-coitus, and the frequency of implantation was calculated as the number of implantation sites per number of embryos transferred. The incidence rates of resorbed and surviving fetuses were calculated as the number of resorptions or surviving fetuses, respectively, per number of implantations. The weights of the surviving fetuses and placentae were measured immediately after dissection.

### 3.8. Immunofluorescent Embryo Stain

Mouse blastocyst cells were fixed by formaldehyde, permeabilized by Triton X-100, blocked by bovine serum albumin (5 mg/mL in PBS), and incubated with anti-Bax or anti-Bcl-2 antibodies (40 mg/mL) at room temperature for 3 h. After washing three times with PBS, embryos were incubated with second antibody conjugated with FITC or Rhodamine (TRITC) (1:100) at room temperature for 1 h and then observed under a fluorescence microscope.

### 3.9. Statistics

The data were analyzed using one-way ANOVA and t-tests and are presented as the mean ± SEM, with significance at P < 0.05.

## 4. Conclusions

In summary, we show for the first time that curcumin induces apoptosis only in the ICM of mouse blastocysts through ROS- and mitochondria-dependent pathways, leading to decreased embryonic development and viability. These findings imply that curcumin is a potential hazardous risk factor for normal embryonic development.

## References

[1] Nadkarni KM Indian Materia Media Popular Prakashan Bombay, India 1976 414 417

[2] Kuttan R Bhanumathy P Nirmala K George MC Potential anticancer activity of turmeric (Curcuma longa) Cancer Lett 1985 29 197 202 4075289 10.1016/0304-3835(85)90159-4

[3] Barthelemy S Vergnes L Moynier M Guyot D Labidalle S Bahraoui E Curcumin and curcumin derivatives inhibit Tat-mediated transactivation of type 1 human immunodeficiency virus long terminal repeat Res. Virol 1998 149 43 52 9561563 10.1016/s0923-2516(97)86899-9

[4] Ramirez-Tortosa MC Mesa MD Aguilera MC Quiles JL Baro L Ramirez-Tortosa CL Martinez-Victoria E Gil A Oral administration of a turmeric extract inhibits LDL oxidation and has hypocholesterolemic effects in rabbits with experimental atherosclerosis Atherosclerosis 1999 147 371 378 10559523 10.1016/s0021-9150(99)00207-5

[5] Ramsewak RS DeWitt DL Nair MG Cytotoxicity, antioxidant and anti-inflammatory activities of curcumins I–III from Curcuma longa Phytomedicine 2000 7 303 308 10969724 10.1016/S0944-7113(00)80048-3

[6] Kim MK Choi GJ Lee HS Fungicidal property of Curcuma longa L. rhizome-derived curcumin against phytopathogenic fungi in a greenhouse J. Agric. Food Chem 2003 51 1578 1581 12617587 10.1021/jf0210369

[7] Reddy RC Vatsala PG Keshamouni VG Padmanaban G Rangarajan PN Curcumin for malaria therapy Biochem. Biophys. Res. Commun 2005 326 472 474 15582601 10.1016/j.bbrc.2004.11.051

[8] Anand P Kunnumakkara AB Newman RA Aggarwal BB Bioavailability of curcumin: Problems and promises Mol. Pharm 2007 4 807 818 17999464 10.1021/mp700113r

[9] Huang MT Lou YR Ma W Newmark HL Reuhl KR Conney AH Inhibitory effects of dietary curcumin on forestomach, duodenal, and colon carcinogenesis in mice Cancer Res 1994 54 5841 5847 7954412

[10] Jiang MC Yang-Yen HF Yen JJ Lin JK Curcumin induces apoptosis in immortalized NIH 3T3 and malignant cancer cell lines Nutr. Cancer 1996 26 111 120 8844727 10.1080/01635589609514468

[11] Jee SH Shen SC Tseng CR Chiu HC Kuo ML Curcumin induces a p53-dependent apoptosis in human basal cell carcinoma cells J. Invest. Dermatol 1998 111 656 661 9764849 10.1046/j.1523-1747.1998.00352.x

[12] Mahmoud NN Carothers AM Grunberger D Bilinski RT Churchill MR Martucci C Newmark HL Bertagnolli MM Plant phenolics decrease intestinal tumors in an animal model of familial adenomatous polyposis Carcinogenesis 2000 21 921 927 10783313 10.1093/carcin/21.5.921

[13] Aggarwal BB Sundaram C Malani N Ichikawa H Curcumin: The Indian solid gold Adv. Exp. Med. Biol 2007 595 1 75 17569205 10.1007/978-0-387-46401-5_1

[14] Khar A Ali AM Pardhasaradhi BV Begum Z Anjum R Antitumor activity of curcumin is mediated through the induction of apoptosis in AK-5 tumor cells FEBS Lett 1999 445 165 168 10069393 10.1016/s0014-5793(99)00114-3

[15] Chan WH Wu HY Chang WH Dosage effects of curcumin on cell death types in a human osteoblast cell line Food Chem. Toxicol 2006 44 1362 1371 16624471 10.1016/j.fct.2006.03.001

[16] Qureshi S Shah AH Ageel AM Toxicity studies on Alpinia galanga and Curcuma longa Planta Med 1992 58 124 127 1529022 10.1055/s-2006-961412

[17] Lao CD Demierre MF Sondak VK Targeting events in melanoma carcinogenesis for the prevention of melanoma Expert Rev. Anticancer Ther 2006 6 1559 1568 17134361 10.1586/14737140.6.11.1559

[18] Lao CD Ruffin MT Normolle D Heath DD Murray SI Bailey JM Boggs ME Crowell J Rock CL Brenner DE Dose escalation of a curcuminoid formulation BMC Compl. Altern. Med 2006 6 10 10.1186/1472-6882-6-10 PMC1434783 16545122

[19] Cheng AL Hsu CH Lin JK Hsu MM Ho YF Shen TS Ko JY Lin JT Lin BR Ming-Shiang W Yu HS Jee SH Chen GS Chen TM Chen CA Lai MK Pu YS Pan MH Wang YJ Tsai CC Hsieh CY Phase I clinical trial of curcumin, a chemopreventive agent, in patients with high-risk or pre-malignant lesions Anticancer Res 2001 21 2895 2900 11712783

[20] Shoba G Joy D Joseph T Majeed M Rajendran R Srinivas PS Influence of piperine on the pharmacokinetics of curcumin in animals and human volunteers Planta Med 1998 64 353 356 9619120 10.1055/s-2006-957450

[21] Hsuuw YD Chang CK Chan WH Yu JS Curcumin prevents methylglyoxal-induced oxidative stress and apoptosis in mouse embryonic stem cells and blastocysts J. Cell. Physiol 2005 205 379 386 15887245 10.1002/jcp.20408

[22] Thompson CB Apoptosis in the pathogenesis and treatment of disease Science 1995 267 1456 1462 7878464 10.1126/science.7878464

[23] Brill A Torchinsky A Carp H Toder V The role of apoptosis in normal and abnormal embryonic development J. Assist. Reprod. Genet 1999 16 512 519 10575578 10.1023/A:1020541019347 PMC3455372

[24] Lotz K Proff P Bienengraeber V Fanghaenel J Gedrange T Weingaertner J Apoptosis as a creative agent of embryonic development of bucca, mentum and nasolacrimal duct. An *in vivo* study in rats J. Craniomaxillofac. Surg 2006 34 Suppl 2 8 13 17071383 10.1016/S1010-5182(06)60003-6

[25] Weingaertner J Proff P Bienengraeber V Gedrange T Fanghaenel J Lotz K *In vivo* study of apoptosis as a creative agent of embryonic development of the primary nasal duct in rats J. Craniomaxillofac. Surg 2006 34 S3 S7 10.1016/S1010-5182(06)60002-4 17071382

[26] Huang FJ Shen CC Chang SY Wu TC Hsuuw YD Retinoic acid decreases the viability of mouse blastocysts *in vitro* Hum. Reprod 2003 18 130 136 12525453 10.1093/humrep/deg018

[27] Chan WH Ginkgolide B induces apoptosis and developmental injury in mouse embryonic stem cells and blastocysts Hum. Reprod 2006 21 2985 2995 16877372 10.1093/humrep/del255

[28] Shang EH Wu RS Aquatic hypoxia is a teratogen and affects fish embryonic development Environ. Sci. Technol 2004 38 4763 4767 15487785 10.1021/es0496423

[29] Detmar J Rabaglino T Taniuchi Y Oh J Acton BM Benito A Nunez G Jurisicova A Embryonic loss due to exposure to polycyclic aromatic hydrocarbons is mediated by Bax Apoptosis 2006 11 1413 1425 16830233 10.1007/s10495-006-8442-3

[30] Chang YJ Chan WH Methylglyoxal has injurious effects on maturation of mouse oocytes, fertilization, and fetal development, via apoptosis Toxicol. Lett 2010 193 217 223 20096340 10.1016/j.toxlet.2010.01.007

[31] Huang FJ Hsuuw YD Lan KC Kang HY Chang SY Hsu YC Huang KE Adverse effects of retinoic acid on embryo development and the selective expression of retinoic acid receptors in mouse blastocysts Hum. Reprod 2006 21 202 209 16199432 10.1093/humrep/dei286

[32] Chan WH Impact of genistein on maturation of mouse oocytes, fertilization, and fetal development Reprod. Toxicol 2009 28 52 58 19490995 10.1016/j.reprotox.2009.03.014

[33] Chan WH Shiao NH Cytotoxic effect of CdSe quantum dots on mouse embryonic development Acta Pharmacol. Sin 2008 29 259 266 18215357 10.1111/j.1745-7254.2008.00743.x

[34] Chan WH Shiao NH Effect of citrinin on mouse embryonic development *in vitro* and *in vivo* Reprod. Toxicol 2007 24 120 125 17572064 10.1016/j.reprotox.2007.04.070

[35] Chan WH Citrinin induces apoptosis via a mitochondria-dependent pathway and inhibition of survival signals in embryonic stem cells, and causes developmental injury in blastocysts Biochem. J 2007 404 317 326 17331071 10.1042/BJ20061875 PMC1868791

[36] Chan WH Citrinin induces apoptosis in mouse embryonic stem cells IUBMB Life 2008 60 171 179 18380009 10.1002/iub.30

[37] Yu F Watts RN Zhang XD Borrow JM Hersey P Involvement of BH3-only proapoptotic proteins in mitochondrial-dependent Phenoxodiol-induced apoptosis of human melanoma cells Anticancer Drugs 2006 17 1151 1161 17075314 10.1097/01.cad.0000231484.17063.9a

[38] Criollo A Galluzzi L Chiara Maiuri M Tasdemir E Lavandero S Kroemer G Mitochondrial control of cell death induced by hyperosmotic stress Apoptosis 2007 1 3 18 17080328 10.1007/s10495-006-0328-x PMC2799004

[39] Bush JA Cheung KJ Jr Li G Curcumin induces apoptosis in human melanoma cells through a Fas receptor/caspase-8 pathway independent of p53 Exp. Cell Res 2001 271 305 314 11716543 10.1006/excr.2001.5381

[40] Kuo ML Huang TS Lin JK Curcumin, an antioxidant and anti-tumor promoter, induces apoptosis in human leukemia cells Biochim. Biophys. Acta 1996 1317 95 100 8950193 10.1016/s0925-4439(96)00032-4

[41] Anto RJ Mukhopadhyay A Denning K Aggarwal BB Curcumin (diferuloylmethane) induces apoptosis through activation of caspase-8, BID cleavage and cytochrome c release: Its suppression by ectopic expression of Bcl-2 and Bcl-xl Carcinogenesis 2002 23 143 150 11756235 10.1093/carcin/23.1.143

[42] Bhaumik S Anjum R Rangaraj N Pardhasaradhi BV Khar A Curcumin mediated apoptosis in AK-5 tumor cells involves the production of reactive oxygen intermediates FEBS Lett 1999 456 311 314 10456330 10.1016/s0014-5793(99)00969-2

[43] Choudhuri T Pal S Agwarwal ML Das T Sa G Curcumin induces apoptosis in human breast cancer cells through p53-dependent Bax induction FEBS Lett 2002 512 334 340 11852106 10.1016/s0014-5793(02)02292-5

[44] Jaruga E Bielak-Zmijewska A Sikora E Skierski J Radziszewska E Piwocka K Bartosz G Glutathione-independent mechanism of apoptosis inhibition by curcumin in rat thymocytes Biochem. Pharmacol 1998 56 961 965 9776306 10.1016/s0006-2952(98)00144-0

[45] Somasundaram S Edmund NA Moore DT Small GW Shi YY Orlowski RZ Dietary curcumin inhibits chemotherapy-induced apoptosis in models of human breast cancer Cancer Res 2002 62 3868 3875 12097302

[46] Cross JC Werb Z Fisher SJ Implantation and the placenta: Key pieces of the development puzzle Science 1994 266 1508 1518 7985020 10.1126/science.7985020

[47] Pampfer S de Hertogh R Vanderheyden I Michiels B Vercheval M Decreased inner cell mass proportion in blastocysts from diabetic rats Diabetes 1990 39 471 476 2318348 10.2337/diab.39.4.471

[48] Kelly SM Robaire B Hales BF Paternal cyclophosphamide treatment causes postimplantation loss via inner cell mass-specific cell death Teratology 1992 45 313 318 1631784 10.1002/tera.1420450310

[49] Tam PP Postimplantation development of mitomycin C-treated mouse blastocysts Teratology 1988 37 205 212 3130676 10.1002/tera.1420370305

[50] Chen CC Chan WH Impact effects of puerarin on mouse embryonic development Reprod. Toxicol 2009 28 530 535 19646524 10.1016/j.reprotox.2009.07.004

[51] Hardy K Cell death in the mammalian blastocyst Mol. Hum. Reprod 1997 3 919 925 9395266 10.1093/molehr/3.10.919

[52] Hardy K Stark J Winston RM Maintenance of the inner cell mass in human blastocysts from fragmented embryos Biol. Reprod 2003 68 1165 1169 12606492 10.1095/biolreprod.102.010090

[53] Byrne AT Southgate J Brison DR Leese HJ Analysis of apoptosis in the preimplantation bovine embryo using TUNEL J. Reprod. Fertil 1999 117 97 105 10645250 10.1530/jrf.0.1170097

[54] Long LH Clement MV Halliwell B Artifacts in cell culture: Rapid generation of hydrogen peroxide on addition of (−)-epigallocatechin, (−)-epigallocatechin gallate, (+)-catechin, and quercetin to commonly used cell culture media Biochem. Biophys. Res. Commun 2000 273 50 53 10873562 10.1006/bbrc.2000.2895

[55] Halliwell B Oxidative stress in cell culture: An under-appreciated problem? FEBS Lett 2003 540 3 6 12681474 10.1016/s0014-5793(03)00235-7

[56] Hardy K Handyside AH Winston RM The human blastocyst: Cell number, death and allocation during late preimplantation development *in vitro* Development 1989 107 597 604 2612378 10.1242/dev.107.3.597

[57] Gardner RL Davies TJ Lack of coupling between onset of giant transformation and genome endoreduplication in the mural trophectoderm of the mouse blastocyst J. Exp. Zool 1993 265 54 60 8459230 10.1002/jez.1402650108

[58] Huang FJ Wu TC Tsai MY Effect of retinoic acid on implantation and post-implantation development of mouse embryos *in vitro* Hum. Reprod 2001 16 2171 2176 11574511 10.1093/humrep/16.10.2171

[59] Witschi E Characterization of developmental stages. Part II. Rat Biology Data Book2nd ed Federation of American Societies of Experimental Biologies Washington, DC, USA 1972 178 180

[60] Armant DR Kaplan HA Lennarz WJ Fibronectin and laminin promote *in vitro* attachment and outgrowth of mouse blastocysts Dev. Biol 1986 116 519 523 3732618 10.1016/0012-1606(86)90152-1

[61] Pampfer S Wuu YD Vanderheyden I De Hertogh R *In vitro* study of the carry-over effect associated with early diabetic embryopathy in the rat Diabetologia 1994 37 855 862 7806014 10.1007/BF00400939

